# Familiarity Is Key: Exploring the Effect of Familiarity on the Face-Voice Correlation

**DOI:** 10.3390/brainsci14020112

**Published:** 2024-01-23

**Authors:** Sarah V. Stevenage, Rebecca Edey, Rebecca Keay, Rebecca Morrison, David J. Robertson

**Affiliations:** 1 School of Psychology, University of Southampton, Southampton SO17 1BJ, UK; re2n18@soton.ac.uk (R.E.); rek1g16@soton.ac.uk (R.K.); rm10g20@soton.ac.uk (R.M.); 2 Department of Psychological Sciences and Health, University of Strathclyde, Glasgow G1 1QE, UK; david.j.robertson@strath.ac.uk

**Keywords:** face processing, voice processing, familiarity, person perception

## Abstract

Recent research has examined the extent to which face and voice processing are associated by virtue of the fact that both tap into a common person perception system. However, existing findings do not yet fully clarify the role of familiarity in this association. Given this, two experiments are presented that examine face-voice correlations for unfamiliar stimuli (Experiment 1) and for familiar stimuli (Experiment 2). With care being taken to use tasks that avoid floor and ceiling effects and that use realistic speech-based voice clips, the results suggested a significant positive but small-sized correlation between face and voice processing when recognizing unfamiliar individuals. In contrast, the correlation when matching familiar individuals was significant and positive, but much larger. The results supported the existing literature suggesting that face and voice processing are aligned as constituents of an overarching person perception system. However, the difference in magnitude of their association here reinforced the view that familiar and unfamiliar stimuli are processed in different ways. This likely reflects the importance of a pre-existing mental representation and cross-talk within the neural architectures when processing familiar faces and voices, and yet the reliance on more superficial stimulus-based and modality-specific analysis when processing unfamiliar faces and voices.

## 1. Introduction

Face processing has been highlighted as an exceptional capability, particularly when stimuli are familiar [1]. Even after a brief exposure, faces can often be recognised, and while changes to appearance can make identification harder, familiarity with a target means that they can often be identified despite such changes [2,3]. In contrast, voice recognition appears to be a more difficult task. In careful laboratory experiments, voices are more likely to elicit familiarity-only reactions or tip-of-the-tongue states [4,5,6], and they are less likely to enable the retrieval of episodic or semantic information about a known speaker [7,8,9]. More recent work using the Famous People Recognition Battery suggested that the accuracy when naming famous people from their faces was 84.4%, while the accuracy when naming from their voices was significantly worse at only 66.0% [10]. Such findings have been used to focus the research effort on accounting for cognitive and neuropsychological differences between face and voice processing (for example, see [11,12]).

More recently, however, discussions are starting to embrace the similarities that might exist between face and voice processing. For instance, the processing of both faces and voices can be understood within the context of a similarity space [13], and the methodologies used to study face processing have been successfully adapted to study voice processing. Furthermore, our theoretical understanding of the task both when recognizing faces and voices now reflects a complex appreciation that the perceiver must overcome within-person variability to tell instances of the same person together and must resolve between-person similarity to tell instances of two different people apart [14,15].

This shift in thinking has been reflected in the early work of Belin et al. [16], who considered the voice as an auditory face providing complementary information about speech, affect, and identity. It has also been captured in the thinking of Yovel and Belin [17], Campanella and Belin [18], and Gainotti [19], who all reflected on the similarity between face and voice processing and the likelihood that initial unimodal processing culminates in the early integrated multimodal processing of identity.

Researchers have recently used a correlational approach to examine the similarity between face and voice processing. Jenkins et al. [20] first examined this using a unique population of super-recognisers with extraordinary abilities to either recognise and/or match faces. Jenkins and colleagues were motivated by the applied question of whether super-face recognisers might also be super-voice recognisers. Using a battery of voice tasks, pre-existing performance data on face tasks, and a generous definition of ‘super’ status (1.5 × SDs above the control population mean), the study showed that most super-voice recognisers were indeed also super-face recognisers (please see Table 1 for a summary of findings). Moreover, across a population that spanned the super to normal populations, face matching ability as measured by the Glasgow Face Matching task (GFMT [21]) correlated positively with voice matching ability (GFMT and BVMT: *r* = 0.30), and face memory as measured by the extended version of the Cambridge Face Memory Task (CFMT+ [22]) correlated positively with voice memory (CFMT+ and GVMT: *r* = 0.12). These results offered an exciting start when considering a correlation between face and voice processing. This being said, the correlations were small to moderate despite the large sample size (*n* = 529), and the inclusion of super-face recognisers could arguably have skewed the results.

Replication with a normal population was required, and three sets of results are of relevance, each of which took pains to match task demands across face and voice processing. First in this regard is the work of Mühl et al. [23], who used the Bangor Voice Matching Task (BVMT) and the Glasgow Face Matching Task (GFMT) to see whether performance on voice and face matching tasks was correlated. They demonstrated a small but significant correlation when matching voices and faces (*r* = 0.24, *n* = 149), a finding which was replicated by Sunilkumar et al. ([24]: *r* = 0.308, *n* = 124) but which failed to reach significance in a smaller test by Johnson, McGettigan, and Lavan ([25]: *r* = 0.20, *n* = 46). Correlations here may be limited by the fact that the BVMT was designed to reveal deficits in voice processing, and thus performance can approach ceiling levels.

Second is the work of Sunilkumar et al. [24], who explored face-voice correlations for memory tasks using the Glasgow Voice Memory Task ([26]: GVMT) and the long version of the Cambridge Face Memory Task (CFMT+). Using a normal participant population, they demonstrated a significant but small correlation between face and voice memory (*r* = 0.259, *n* = 95). While these results aligned with the significant correlations described above, the use of identical vowel sounds in the GVMT at the study and test stages may suggest some caution when interpreting these findings.

**Table 1:** Summary of the number of super-voice recognisers as measured on each of the tasks reported by Jenkins et al. [20] according to their super-face recogniser status.

	BVMT (Mühl et al. [23])	GVMT (Aglieri et al. [26])	Famous Voice Recognition
Also Super Face Recognisers	56	50	47
Not Super Face Recognisers	15	22	24
Total no. Super Voice Recognisers	71	72	71

Interestingly, Sunilkumar et al. [24] addressed the issue of stimulus realism by exploring face-voice correlations when more naturalistic speech samples were utilised. Both in face-voice matching and face-voice memory, correlations became stronger when more naturalistic voice clips were used in place of the syllables of the BVMT and the identical vowel sounds of the GVMT. In order to avoid ceiling effects when processing these naturalistic voice clips, acoustic modifications were applied to simulate speech in a cave or aircraft hangar, or with echo, radio, telephone noise or gargle applied. Whilst encouraging, these results perhaps still beg the question of whether face-voice correlations may be reliably obtained in normal populations and with everyday speech samples.

Last in this regard is the work of Johnson et al. [25]. As well as using the matching tasks noted above, Johnson et al. used a face sorting task and a voice sorting task with multiple naturally varying instances of the faces and voices of two unfamiliar male Canadian actors. With a modest sample size, Johnson et al. nevertheless revealed a significant correlation when sorting faces and voices (Kendall’s tau = 0.27, *n* = 46). This task arguably represents the most elegant set of results, drawing as it does on realistic stimuli, a normal participant population, and a realistic telling together/telling apart sorting task. The correlation is nevertheless small and perhaps rests on the ability to tell stimuli apart, which itself is a task that approached ceiling levels of performance. Additionally, the results rested on the performance of English participants listening to Canadian voices, and thus there was the potential for accent effects to influence performance in the voice sorting task.

For two reasons, methodological issues perhaps render the existing examination of face-voice correlations incomplete. First, studies have tended to use stimuli, which are either limited in number or in ecological validity. These concerns may lead one to question the generalisability of existing results to more naturalistic stimuli and contexts. Given that some of the previous findings indicated only very weak correlations, it seems prudent to replicate these results with improved stimuli and methods. Second, studies have concentrated almost exclusively on the processing of unfamiliar stimuli (although see Jenkins et al. [20] for correlations between unfamiliar face processing and familiar voice processing). As such, the studies have largely ignored the differences between familiar and unfamiliar processing of faces [14,27] and voices [28,29]. This is perhaps an important oversight because such consideration may contribute to an understanding of whether similarities between face and voice processing reflect modality-general processes, or separate parallel modality specific processes [25]. The present paper will address both issues using methodologies that utilise naturalistic stimuli and tasks which avoid both floor and ceiling effects.

The purpose of Experiment 1 was to replicate existing demonstrations of a face-voice correlation using realistic samples of unfamiliar stimuli. Strength is provided through the use of careful methodologies which avoid floor and ceiling effects, as well as through the use of more ecologically valid stimuli, which support a stronger generalisability of results. In addition, the present paper extends the previous consideration of a face-voice correlation by exploring the important but as yet untested question of a face-voice correlation with familiar stimuli (Experiment 2). In this regard, the present paper offers novelty in its empirical focus whilst also contributing to more theoretical issues concerning the differences when processing unfamiliar and familiar stimuli.

## 2. Experiment 1

The question addressed by Experiment 1 was whether the face-voice correlation demonstrated in the literature is robust when tested with naturalistic stimuli and methods that avoid floor and ceiling effects. On the basis of previous studies with super-recognisers [20], and subsequent studies with normal listeners [23,24,25] a face-voice correlation may be likely albeit small in magnitude. However, according to Johnson et al. [25] this face-voice correlation may only emerge in the general population when task demands are well-aligned. This said, the use of CVC stimuli, vowel-based stimuli, acoustically modified stimuli, or other-accent stimuli may weaken the validity of previous demonstrations, justifying a re-examination here with new stimuli. Experiment 1 addresses this issue.

## 3. Materials and Methods for Experiment 1

### 3.1. Design

A within-participants design was used in which participants completed an unfamiliar face matching task (the Glasgow Face Matching Test-short version [GFMT]) and a bespoke unfamiliar voice matching task. This was used rather than a standardised voice matching task, given the desire to measure voice processing using stimuli that went beyond short vowel sounds. Accuracy was scored in the published way for the GFMT to allow comparison to published norms. This yielded an overall score (out of 40). For comparability, the unfamiliar voice matching task was also scored to yield an overall accuracy score (out of 40). Both scores were converted to proportions for the purposes of analysis.

### 3.2. Participants

Based on the literature, the correlation between face and voice processing is expected to be small when stimuli are unfamiliar. Power Analysis using G*Power 3.1.9.7 suggested that a minimum of 153 participants would be required to demonstrate a 1-tailed small correlation (0.2—the smallest of the published correlations in tests of normal listeners) given a power of 80% and an alpha of 0.05. We exceeded this number, with a total of 161 participants (106 females, 54 males, 1 undeclared) who took part on a volunteer basis or in return for course credit or Prolific payment. Ages ranged from 18 to 53 years (*M* = 21.8, *SD* = 5.50). Participants were all native UK English speakers, and self-report confirmed normal or corrected hearing and vision. A subset of 71 engaged participants were recruited via the online recruitment platform Prolific, whereas the remainder were recruited from a university population and were tested in-person.

### 3.3. Materials

#### 3.3.1. Unfamiliar Faces

The GFMT-short involved the presentation of a standardised set of stimuli as described in the literature (GFMT: [21]). Specifically, the GFMT-short employed 40 pairs of faces, presented as greyscale photographs at a full-face pose only and with neutral expressions. Hair was visible, but all details below the jawline were removed. Half of the GFMT trials represented two different images of the ‘same’ individual, whilst the remaining GFMT trials represented two ‘different’ individuals matched for sex. The task was to indicate whether the two images represented the ‘same’ individual or ‘different’ individuals.

#### 3.3.2. Unfamiliar Voices

The Unfamiliar Voice Matching Task used 40 pairs of voice clips selected from a larger set of stimuli collected as part of the Superidentity Project. All speakers were free from audible speech impediments and spoke with a standard southern British accent. In all voice clips, the speaker uttered a single sentence lasting 4–5 s in duration. Half of the trials utilised two different speech clips from the ‘same’ individual, whilst the remaining trials utilised two different speech clips from ‘different’ individuals. Care was taken with foil selection on ‘different trials,’ with each foil being selected based on its similarity to each target (as determined by experimenter listening). This ensured that the trials were not too easy. In addition, care was taken to counterbalance across participants the identity of the speakers in ‘same’ and ‘different’ trials in order to avoid item effects. This necessitated 2 clips of 40 target speakers (20 male, 20 female), and 1 clip of 20 foil speakers (10 male, 10 female). As with the GFMT, the task was to indicate whether the two samples came from the ‘same’ individual or from ‘different’ individuals.

The tasks were programmed within iSurvey and were presented on a Dell Precision M4600 laptop (Dell, London, UK), or the Prolific participants’ own laptop. Participants listened to the sound clips via the computer speakers set to an audible but adjustable level having completed an audio check as part of the pilot phase.

### 3.4. Procedure

**Figure 1:**
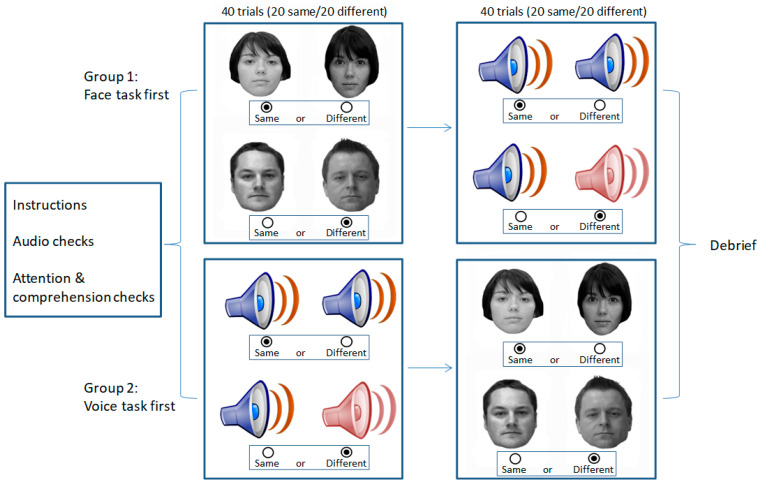
Illustration of the method for Experiment 1 in which participants completed a face matching task (GFMT) and a voice matching task with order of tasks counterbalanced. Each task consisted of 40 trials (20 same, 20 different) and performance was calculated as the proportion of correct responses.

Participants were either tested individually in a quiet location, or completed the study remotely, with appropriate attention and comprehension checks in place. Informed consent was obtained from all subjects involved in the study. In both participant groups, care was taken to counterbalance the order of tasks across participants and to provide self-paced rest breaks between tasks. This notwithstanding, the procedure for each task was identical across participants, as follows and as illustrated in Figure 1.

In the GFMT-short version task [21], two unfamiliar faces were presented side by side. Participants were asked to identify whether the two faces were the ‘same’ or ‘different’ by clicking on the appropriate onscreen buttons. Images remained in view until participant response, and accuracy of response was recorded across 40 trials. This thus represented a face matching task with unfamiliar faces.

In the unfamiliar voice matching task, participants were presented with a practice phase followed by a set of sequential voice matching trials. In the practice phase, participants completed 10 trials in which they were asked to indicate whether two successively presented audible beeps were of the ‘same pitch’ or of ‘different pitches’. This thus required the audio to be working and required the participants to discriminate between the two sounds, mimicking the main task. Participants responded by clicking on the appropriate on-screen response button. Following these practice trials, participants completed a set of 40 experimental trials that involved the presentation of two speech clips separated by a 5 s gap. Participants used the onscreen buttons to indicate whether the two clips were uttered by the ‘same’ speaker or by ‘different’ speakers, and accuracy was recorded.

Completion of the face and voice tasks took about 30 min in one sitting. Following this, participants were thanked and debriefed.

## 4. Results and Discussion for Experiment 1

### 4.1. Scoring

**Figure 2:**
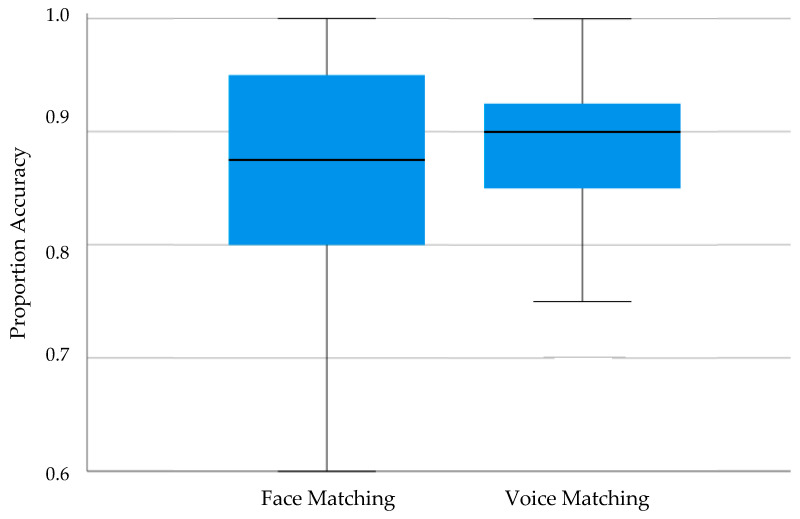
Boxplot of proportion(accuracy) when matching unfamiliar faces on the GFMT and when matching unfamiliar voices in Experiment 1.

Traditional scoring of accuracy was used both for the GFMT (out of 40) and for the Unfamiliar Voice Matching task (out of 40). The data from one male participant were dropped for failure to complete one task. In addition, a further three male participants were excluded as analysis revealed scores below 60% suggesting a lack of engagement either on the GFMT task (two participants) or on the Unfamiliar Voice Matching Task (one participant). Performance across the remaining participants is summarized in Figure 2 below.

Across the remaining participants, independent samples *t*-tests confirmed no effect of order of tasks for either the GFMT (*t*_(155)_ = 0.976, *p* = 0.330, Cohen’s *d* = 0.156) or for the Unfamiliar Voice Matching task (*t*_(155)_ = 0.708, *p* = 0.480, Cohen’s *d* = 0.113). Similarly, there was no difference in performance across the participants tested online versus those tested in-person for either the GFMT (*t*_(155)_ = 0.007, *p* = 0.995, Cohen’s *d* = 0.001) or for the Unfamiliar Voice Matching task (*t*_(155)_ = 0.838, *p* = 0.404, Cohen’s *d* = 0.136). As a result, all further analyses were conducted having collapsed across these two variables.

In examining performance, a series of one-sample *t*-tests first compared performance to chance levels for each task. This revealed performance levels significantly above that associated with guessing alone for each task (GFMT: Mean accuracy = 0.86, *t*_(156)_ = 49.83, *p* < 0.001, Cohen’s *d* = 3.98; Unfamiliar Voice Matching: Mean accuracy = 0.89, *t*_(156)_ = 79.97, *p* < 0.001, Cohen’s *d* = 6.38). Moreover, neither task showed any indication of ceiling effects (GFMT: *t*_(156)_ = 18.84, *p* < 0.001, Cohen’s *d* = 1.50; Unfamiliar Voice Matching: *t*_(156)_ = 23.15, *p* < 0.001, Cohen’s *d* = 1.85). Finally, the participants in Experiment 1 performed marginally better on the GFMT relative to the published norm of 0.813 (*t*_(156)_ = 6.84, *p* < 0.001, Cohen’s *d* = 0.55). These results suggested that the tasks were neither too easy nor too difficult whilst also showing participants’ engagement as revealed on the GFMT.

### 4.2. Correlation of Unfamiliar Face and Voice Processing

A Pearson’s bivariate correlation was used to explore the association between performance on the unfamiliar face matching task, and the unfamiliar voice matching task. This revealed a significant positive but weak correlation between performance on the two tasks (*r*_(157)_ = 0.143, *p*_(1-tailed)_ = 0.037) which reached significance only as a one-tailed test. As such, a significant, positive, yet small correlation was revealed here when matching unfamiliar faces and voices, and when using naturalistic stimuli and tasks that avoided ceiling and floor effects.

## 5. Experiment 2

Experiment 2 provided an examination of face-voice correlations for famous stimuli. An improved ‘Before They Were Famous’ (BTWF: [22]) task was utilised, alongside a Celebrity Voice Recognition task that used free speech clips to address ecological validity. Importantly, and noting Johnson et al.’s [25] emphasis on common task demands, the use of a recognition task facilitated straightforward and direct comparison of performance across modalities. Given the theoretical expectations around a similarity between face and voice processing, a positive correlation between face and voice recognition abilities was predicted. Moreover, given the parallels that might exist through common high-level person identity processing, the correlation between familiar face and voice processing may be expected to be larger than that with unfamiliar stimuli. However, the magnitude of this correlation remains a point of empirical investigation.

## 6. Materials and Methods for Experiment 2

### 6.1. Design

A within-participants design was used in which participants completed both a celebrity face recognition task and a celebrity voice recognition task. Conditionalised naming scores were calculated for both tasks and represented the dependent variables.

### 6.2. Participants

Power Analysis using G*Power 3.1.9.7 suggested that a minimum of 67 participants would be required to demonstrate a one-tailed medium correlation (0.3) given a power of 80% and an alpha of 0.05. The sample size here exceeded this number. A total of 96 participants (76 females, 19 males, 1 non-declared) took part on a volunteer basis or in return for course credit. Ages ranged from 18 to 53 years (*M* = 20.6, *SD* = 3.89) and participants were all native UK English speakers. In addition, participants self-reported normal or corrected hearing and vision together with a good awareness of celebrities in the public eye reducing the risk of floor effects.

### 6.3. Materials

#### 6.3.1. Celebrity Faces

A total of 28 celebrities (18 males) were selected from a larger pool of individuals, all of whom were A-list celebrities in the public eye in the US or the UK through stage, screen, or music. Prior ratings by a set of 34 judges (drawn from the same pool as the participants) confirmed that the celebrities were familiar by name, as shown through familiarity ratings on a 1–7 scale (1 = unfamiliar, 2–7 = levels of familiarity rising to 7 = very familiar indeed). Using this familiarity rating scale, the selected set of 28 celebrities received a mean familiarity of 5.77 (*SD* = 1.44, min = 2.5, max = 7). Additional selection criteria specified that all celebrities were Caucasian (to remove any other-race face effects for our Caucasian participants) and that a suitable image of the celebrity as a child could be obtained.

The childhood images of the celebrities were very carefully chosen. They were obtained via internet searches but were constrained to depict the celebrity in a good-quality image (minimum 250 × 250 pixels) and aged between 6 and 12 years of age. Celebrity age was estimated by the experimenters through the visual examination of teeth within the smiling images. Images were selected that showed adult upper front teeth (emerging at roughly age 6), but 12-year-old molars were unlikely to be present as judged by jaw definition. These constraints sought to provide greater control across the set of items regarding the similarity in age and thus in appearance between childhood and adult images in a way that perhaps improved on previous iterations of the BTWF task. Once obtained, all childhood images were pre-processed using PAINT to standardise the image size to approximately 9.5 × 9.5 cm. The majority of celebrities were presented as colour images, and all were presented within their original backgrounds. In all cases, background did not give clues as to celebrity identity.

#### 6.3.2. Celebrity Voices

A total of 28 celebrities (21 males) were selected, with only one of these celebrities being used in both the face task and the voice task. (The duplication of one celebrity across the BTWF face task and the Celebrity Voice naming task was unfortunate but was necessitated as a product of the strict selection criteria and thus the limit on the number of stimuli meeting the criteria. There was no indication from participant comments that they noticed the duplication of this individual across tasks.) Again, the celebrities were A-list celebrities in the public eye through stage, screen, or music. They were selected as a result of their familiarity with a second set of 22 judges (drawn from the same population as the participants). Using the same familiarity rating scale described above, the celebrity speakers received a mean rating of 5.35 out of 7 (*SD* = 0.64, min = 3.64, max = 6.41). The 28 celebrity speakers all spoke with a British English accent, minimizing the potential for other-accent effects to be experienced by the British participants. The speakers did, however, vary in regional accent and thus in rated distinctiveness as judged by the 22 judges (*M* = 3.97, *SD* = 1.13, min = 1.67, max = 6.29 on a 7-point scale in which 1 = not at all distinctive and 7 = very distinctive indeed). Rather than controlling vocal distinctiveness, this variation was considered desirable and representative of the range of voices one knows and encounters in real life.

For each of the 28 celebrities, a free-speech clip was obtained by extracting a segment of uninterrupted speech from YouTube sources such as interviews or chat shows. Care was taken to ensure that the content of the speech did not reveal the identity of the speaker and that no other voices or background noise masked the celebrity voice. Speech segments were edited using Audacity 3.1.0 to create 8 s samples. As such, clip length was standardised but the end of the speech sample did not always coincide with the end of a sentence or phrase.

### 6.4. Procedure

Following the provision of informed consent, participants completed the study in person via iSurvey. They were instructed to complete the study in one sitting, using personal headphones if available, and ensuring a quiet environment in which there could be no collusion or distraction. Participants were also advised of the likely overall study time, and their progress through the study in order to encourage continued engagement.

**Figure 3:**
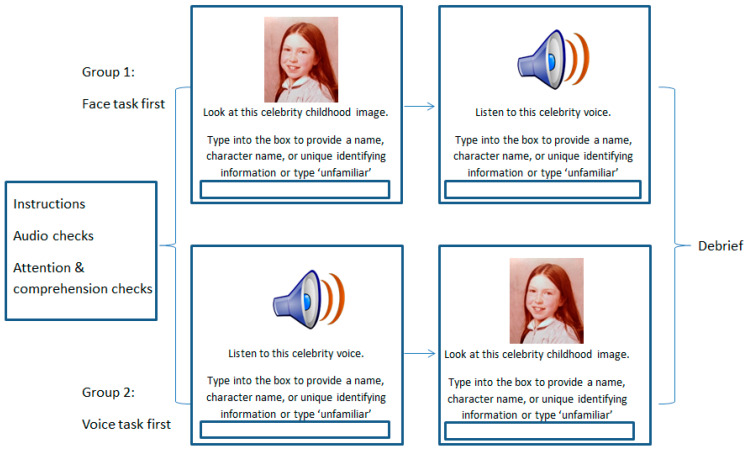
Illustration of the method for Experiment 2 in which participants completed a Celebrity face naming task (BTWF) and a Celebrity voice naming task with order of tasks counterbalanced. Given copyright issues with the reproduction of celebrity images, the image displayed is a childhood image of the first author for illustrative purposes only.

Participants were randomly assigned to one of two groups which differed only in the order of the face and voice tasks. This aside, the tasks were identical, and are illustrated in Figure 3.

The Celebrity Voice Recognition task involved the presentation of each voice sample one at a time in a random order. Participants were advised that all speakers were drawn from the British stage, screen, or music industry in order to constrain the likely search set. Participants were asked to listen to the voice sample and then identify the speaker either by typing their name, their character name, or any unique identifying information into an on-screen text box. Participants could play the voice as often as they wished before providing their answer and there was no time constraint within which they had to respond. If a voice was unfamiliar to the participant, they were instructed to enter ‘unfamiliar’ into the text box and the next voice was presented.

The BTWF task consisted of the presentation of a childhood image of a current A-list celebrity. In this task, participants were advised that all celebrities were drawn from the British and American stage, screen, or music industries, again allowing the search set to be defined. Participants were then presented with the childhood image of each of the 28 celebrities, one at a time, with the image remaining on screen until participant response. Participants were asked to study each image and identify the celebrity by again typing their name, character name, or unique identifying piece of information into the on-screen text box. As before, if a face was unfamiliar to the participant, they entered ‘unfamiliar’ into the text box before moving on to the next childhood image.

Finally, participants completed a familiarity check with all celebrities. This was achieved by asking participants to rate the familiarity of each celebrity as indicated by their name. A 7-point rating scale was used, in which a rating of 1 indicated that the celebrity was unknown and a rating of 2–7 indicated a rising level of familiarity. Importantly, participants could not backtrack to the face task or the voice task and thus could not use the names within the familiarity check to assist them in the previous tasks. If any of the celebrities received a rating of 1 in the familiarity check, that voice or face was removed from the previous recognition task as there could be no expectation that the individual could be recognised. This enabled the calculation of Conditionalised Naming Scores on both tasks. The entire study lasted between 30 and 40 min, following which participants were thanked and debriefed.

## 7. Results and Discussion for Experiment 2

At the outset, the data from three participants were excluded from analysis either because they completed only one of the two tasks (*n* = 2) or because the familiarity check revealed that they were unfamiliar with too many celebrities to enable completion of the tasks (*n* = 1). Examination of the Conditionalised Naming Scores in face and voice tasks from the remaining 93 participants (73 females, 19 males, 1 non-declared) revealed no outliers, thus no other participants were excluded. The use of an in-person testing procedure, together with a check of task completion times (relative to known completion times) provided reassurance that performance on both tasks reflected participants’ true abilities.

**Figure 4:**
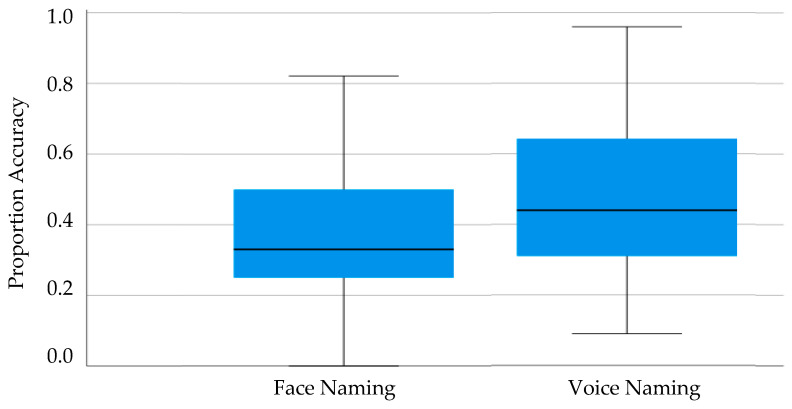
Boxplot of Conditionalised Naming Scores when naming childhood images of celebrity faces (BTWF task) and when naming celebrity voices in Experiment 2.

Preliminary analysis confirmed no effect of task order on either the BTWF task (*t*_(91)_ < 0.73, *p* = 0.47, Cohen’s *d* = 0.15) or the Celebrity Voice Recognition task (*t*_(91)_ = 0.41, *p* = 0.68, Cohen’s *d* = 0.09) enabling all subsequent analyses to be collapsed across this variable. The conditionalised naming scores are summarized in Figure 4 for each participant group and each task.

Analysis by means of one-sample *t*-tests confirmed that performance was above zero for both the BTWF task (*t*_(92)_ = 19.02, *p* < 0.001, Cohen’s *d* = 1.97) and the Celebrity Voice Recognition task (*t*_(92)_ = 21.35, *p* < 0.001, Cohen’s *d* = 2.21).

Having demonstrated that the face and voice identification tasks were possible, the correlation between famous face and voice identification was examined. Pearson’s bivariate correlation revealed a significant, positive, and moderately-sized association between face and voice identification (*r*_(93)_ = 0.568, *p* < 0.001). As such, the capacity for face identification and voice identification were shown to be associated when stimuli were familiar.

## 8. Discussion

The purpose of the present paper was to explore the possibility that similarities may exist in the processing of face and voice identity such that an individual who is good at one task may also be good at the other task. Previous demonstrations of a correlation in performance have used either super-recognisers as participants [20] or have used normal listeners but stimuli which are limited to CVC stimuli [23,24,25], identical vowel sounds [24] acoustically modified speech clips [24], or a task which risked ceiling effects or other-accent effects [25]. The opportunity thus existed to revisit the face-voice correlation using improved stimuli and tasks with a view to replicating existing face-voice correlations with unfamiliar stimuli. Moreover, the opportunity existed to extend the exploration of this correlation to familiar stimuli.

Experiment 1 used a matching task with unfamiliar faces and voices and revealed a small but significant correlation, as anticipated. As such, these results confirmed previous demonstrations of a correlation when processing unfamiliar faces and voices. However, methods were used here that avoided both floor and ceiling effects, and stimuli were used that had realism whilst avoiding potential accent effects. The correlation between face and voice matching speaks to the theoretical position of Belin et al. [16], who considered the voice to provide complementary information to the face, potentially by being processed in a complementary fashion. This said, the demonstration of a correlation itself cannot address the basis of the similarity between face and voice processing. Specifically, it cannot determine whether that similarity rested on the involvement of common modality-general processes or the existence of similar parallel modality-specific processes [25].

The results of Experiment 2 again explored the existence of a face-voice correlation, but this time for familiar stimuli. Using identification tasks that appropriately avoided the possibility of floor and ceiling effects, participants were asked to name familiar celebrities from either their face or their voice. The results again revealed a significant correlation between face and voice processing, and this alone is noteworthy as perhaps the first demonstration of its kind. However, more important is the magnitude of this correlation, which considerably outstripped the correlation evident when stimuli were unfamiliar.

### 8.1. Theoretical Considerations Underpinning Face-Voice Correlations

In accounting for the greater correlation of face and voice processing when stimuli were familiar, two factors may be important. First, there is the influence of semantic information associated with familiar people, the retrieval of which can in turn improve activation at face and voice recognition units through the influence of bi-directional links [19]. This top-down influence consequently contributes to the unimodal processing both of faces (in the Fusiform Face area [FFA] and the Occipital Face Area [OFA]) and of voices (in the temporal voice area (TVA: [30]) providing an opportunity for each to influence the other. A correlation between familiar face and voice processing would naturally result.

Second, there is now a wealth of evidence to suggest the existence of early integration of activation between brain areas associated with familiar face and voice processing. Two empirical demonstrations illustrate this notion. First, Robertson and Schweinberger [31] noted that the co-presentation of a voice alongside the corresponding dynamic face facilitated familiar voice recognition, whereas the co-presentation of a voice alongside a different dynamic face impaired familiar voice recognition. The same pattern of face-voice integration was not present when stimuli were unfamiliar, suggesting that this face-voice integration appeared to depend on familiarity. Second, Zäske et al. [32] drew on the demonstration of contrastive perceptual aftereffects. A contrastive aftereffect occurs when a morphed (identity-ambiguous) voice is less likely to be identified as a given speaker if the participant has previously been adapted to that speaker. Of particular interest here is that this contrastive vocal aftereffect could be demonstrated following adaptation to the face as well as following adaptation to the voice, again suggesting face-voice integration. As with the example of facilitated recognition above, this demonstration of cross-modal contrastive aftereffects was evident when stimuli were familiar but not when unfamiliar.

These empirical examples of face-voice integration may rest on the compelling demonstration of neural cross-talk when processing familiar faces and voices. This cross-talk means that the presentation of a known voice may trigger activation in the face areas of the brain, and vice versa. A face-voice correlation would follow as a natural consequence of such cross-talk. To illustrate, von Kriegstein et al. [33,34] noted that familiar voices can trigger activation in the fusiform face area (FFA), usually activated by faces. Moreover, this cross-talk likely underpins the empirical demonstration of voice-face priming noted by Blank et al. [35]. More compelling is the demonstration of structural linkage between face and voice areas as revealed using probabilistic tractography to visualise fibre tracts [36].

Adding a further perspective to the dialogue regarding face-voice integration, evidence now has emerged to support the existence of areas of the brain which integrate faces and voices into a unique multimodal percept [36,37] which sit alongside the neurally linked unimodal percepts. Taken together, these empirical and neurological demonstrations consequently suggest that we should expect a correlation between familiar face and voice processing as a natural consequence of face-voice integration, cross-modal neural activation, and the generation of multimodal percepts when stimuli are familiar.

If one then thinks of familiar face and voice processing as resulting from unimodal processing plus the beneficial impact of face-voice integration and neural cross-talk, then it may not be surprising that the correlation between familiar face and voice processing is greater than that for unfamiliar stimuli. Given this train of thought, one might then justifiably ask about the basis for the unfamiliar face and voice correlation seen in previous published examples and seen here in Experiment 1. As Johnson et al. [25] note, it is possible that the small correlations witnessed are the result of similarity of task demands rather than being the result of a true similarity between face and voice processing per se. They make this suggestion based on the greater correlation revealed when task demands were strongly aligned by using a sorting task rather than a matching task for unfamiliar face and voice processing. This said, it is notable that a similarity of task demands cannot solely be responsible for a correlation in unfamiliar face/voice performance: certainly, face memory correlated with voice memory as measured by the Glasgow Voice Memory Task-voices, but not with the memory for bell sounds also measured by the Glasgow Voice Memory Task-bells using exactly the same task demands [20].

With this in mind, it is possible that the face-voice correlation for unfamiliar stimuli rests instead on the similarity of unimodal processing when telling faces together and apart, and when telling voices together and apart. Both processes can be explained nicely within a theoretical similarity space framework [13] in which identities are represented as regions (rather than points) in space. This framework allows description of the mapping together of instances of the same person despite variability, and the mapping apart of instances of different people despite similarity (see [15]). Indeed, Johnson et al. [25] offer tentative evidence to suggest that it is the ability to map apart instances of different faces and voices which may underpin their small correlation between unfamiliar face and voice processing. This said, familiar and unfamiliar stimuli attract quite different levels of performance when telling instances together and apart, as noted in sorting tasks (for example, see [38]). Hancock [1] notes that this is perhaps not surprising as the task demands differ in a qualitative fashion. When stimuli are familiar, the question is whether the faces represent multiple instances of the same known person, and performance benefits from the existence of a stored representation. In contrast, when stimuli are unfamiliar, task performance depends on a superficial match of features, which is rendered more or less easy depending on the similarity of the instances themselves. In this sense, Hancock highlights that when processing unfamiliar stimuli, whether faces or voices, the perceiver is largely reliant on a superficial short term pattern matching strategy with potential reference to some rapidly formed mental representation. Any correlation between unfamiliar face and voice processing may thus rest on the similarity of task demands combined with the reliance on corresponding short term perceptual comparisons.

### 8.2. A Note on the Influence of Participant Demographics

Notable when considering these results is the fact that the data were obtained predominantly from a student population of young, healthy adults. On this basis, one may question the generalisability of the findings outlined here. In this regard, it is useful to note that the performance of the student population did not differ from that of the engaged Prolific population in Experiment 1, and this may go some way towards providing reassurance on the generalisability of results. Nevertheless, both populations are perhaps familiar with the completion of experimental studies and consequently may have a high motivation to excel even in challenging tasks. Indeed, the participants in Experiment 1 performed better than published norms in the GFMT task where benchmark data exist. This said, the use of a normal (rather than super) population of participants was purposeful in the current work, ensuring that performance was more representative of the general population.

It is worth noting, however, that differences in the strength of face-voice correlations might be expected in younger populations as they acquire expertise with both faces and voices. Likewise, differences might be expected in older populations given any differential impact of cognitive decline across face and voice tasks, given any differential impact of both hearing and vision problems, and the engagement with any corrective mechanisms such as glasses or hearing aids. Future work would be well directed to examine the strength of face-voice correlations across the lifespan, but this was beyond the scope of the current work.

### 8.3. Implications

Given the current demonstration of a small but significant correlation between unfamiliar face and voice processing and a larger, more significant correlation between familiar face and voice processing, it is prudent to consider what is gained from this demonstration. First and foremost, this demonstration reminds us of the distinction between familiar and unfamiliar processing, as highlighted in faces by Megreya and Burton [27] and as highlighted in voices by van Lancker and Kreiman [39] and Kreiman and Sidtis [28] (see [29] for a review). Second, the present results may contribute to current discussions surrounding the existence of a common modality-free person perception factor ‘*p*’ that provides a basis for the common high-level processing of familiar faces and voices (see [24]). Finally, the present results contribute to an applied question regarding the reliability of face and voice processing by allowing us to conclude that perceivers who are ‘good with faces’ are also likely to be ‘good with voices’ particularly when stimuli are familiar.

## 9. Conclusions and Future Work

The present paper has used realistic stimuli and methods that avoid floor and ceiling effects. The results revealed a correlation between unfamiliar faces and voice processing and a greater correlation between familiar face and voice processing. These findings accord with predictions if one assumes that similar task demands and parallel unimodal processing may drive the small effects seen with unfamiliar stimuli, and the benefits of face-voice integration and neural cross-talk additionally contribute to drive the larger effects seen with familiar stimuli. As is often the case with voice processing [40,41,42], it may be wise to replicate the current findings regarding a familiar face-voice correlation using both personally familiar stimuli and newly learned stimuli in order to ensure that any effects are not subject to biases that could result from the fact that celebrity faces are often encountered more than celebrity voices. Similarly, it would be wise to follow the interesting questions laid down by Kreiman and Sidtis [28] regarding the development of familiarity. Indeed, with voice recognition being the harder task compared to face recognition (see [6]), the rate at which familiarity is acquired may differ substantially across faces and voices, leading to a complex set of relationships across unimodal yet linked brain regions. What is clear is that a correlation between face and voice processing does exist, and the demonstration here of the importance of familiarity serves as a reminder that the processes underpinning face and voice processing remain intricate yet intertwined.

## Data Availability

The data that support the analysis in this paper are available within Supplemental Materials.

## Supplementary Materials

The following supporting information can be downloaded at: https://www.mdpi.com/article/10.3390/brainsci14020112/s1, Files S1: Excel datafile.
